# Parameters That Can Be Used to Quantify Reduction Accuracy in Talar Neck Fractures and Malunions: A PRISMA-Compliant Scoping Review and Meta-Analysis

**DOI:** 10.7759/cureus.58161

**Published:** 2024-04-13

**Authors:** Siddhartha Sharma, Karan Jindal, Sandeep Patel, Sharad Prabhkar, Mahesh Prakash, Stefan Rammelt, Mandeep Dhillon

**Affiliations:** 1 Foot and Ankle Biomechanics, Experimentation and Research Laboratory, Department of Orthopaedics, Post Graduate Institute of Medical Education and Research, Chandigarh, Chandigarh, IND; 2 Orthopedics, Dr. B. R. Ambedkar State Institute of Medical Sciences, Mohali, IND; 3 Foot and Ankle Biomechanics, Experimentation and Research Laboratory, Department of Orthopaedics, Post Graduate Institute of Medical Education and Research, Chandigarh, IND; 4 Radiology, Post Graduate Institute of Medical Education and Research, Chandigarh, IND; 5 Orthopedics, Accident, and Plastic Surgery, UniversitätsCentrum für Orthopädie, Unfall- und Plastische Chirurgie, University Hospital Carl Gustav Carus, Dresden, DEU

**Keywords:** meta-analysis, systematic scoping review, talar declination angle, talar torsion, talar inclination angle, morphometry, geometry, reduction accuracy, talus malunion, talus fracture

## Abstract

Understanding the three-dimensional anatomy of the talar neck is essential in assessing the accuracy of reduction in talar neck fractures as well as for planning surgical correction for talar malunions. However, the geometrical parameters that describe this anatomy are sparsely reported in the orthopedics literature. We aimed to identify from the existing literature, geometrical parameters that describe the anatomy of the talar neck, determine how these are measured, and their normative values. A scoping literature review was conducted in accordance with the Preferred Reporting Items for Systematic Reviews and Meta-Analyses (PRISMA) extension for scoping reviews (PRISMA-ScR) guidelines. The primary searches were conducted on the PubMed, Embase, and Scopus databases. Any original research study looking at the human talus neck geometry was included. Parameters that described the anatomy of the talar neck were identified, and pooled estimates were determined by the random-effects meta-analysis model. Heterogeneity was assessed by the *I^2^* test and leave-one-out meta-analysis. Subgroup analysis was done to compare the values of parameters between the Asian and Non-Asian populations. The risk of bias was assessed by the National Institutes of Health (NIH) Case Series Tool. The combined searches yielded 6326 results, of which 21 studies were included in the review and 15 in six different sets of metanalysis. The majority of the studies (n=19, 90.5%) evaluated adult tali, and only two (9.5%) evaluated pediatric tali. In most of the studies (n=13, 61.9%), talus neck geometry was evaluated on dry bones or anatomical specimens; evaluation by imaging techniques (radiographs, CT, MRI, and radiostereometric analysis) was used in eight studies, (39.1%). A total of eight different geometrical parameters (neck length, height, width, declination angle, inclination angle, torsion angle, circumference, and cross-sectional area) were identified. Except for talar torsion, variability was noted in methods of measurement of all other parameters. Subgroup analysis revealed that Asians had a higher neck height as compared to non-Asians; other parameters were not significantly different. Although the literature reports geometrical parameters to assess the talar geometry, the methods of measurement of these parameters are variable. Most of the available literature describes measurement techniques on cadaveric tali, and there is no literature on how these parameters should be measured on conventional CT or MRI slices. Further research needs to focus on the standardization of measurement techniques for these parameters on conventional CT and/or MRI scans.

## Introduction and background

The talus is the second-largest tarsal bone, and participates in the formation of three essential lower limb joints, i.e., the ankle (talocrural), subtalar, and the talonavicular joints. The talar neck connects the talar body to the talar head, and is instrumental in load transmission from the tibia to the midfoot as well as the hindfoot [1]. Alterations in talar neck geometry, which may occur after trauma, or as a result of congenital conditions, can result in altered motion as well as load transmission across these joints, and result in pain and premature arthrosis [2,3]. Hence, an understanding of the three-dimensional geometry of the talar neck is vital in judging reduction after operative fixation of talar neck fractures, as well as in planning corrective surgery for talar malunions. However, the parameters that describe the three-dimensional anatomy of the talar neck are sparsely reported in the literature, and there is no consensus on which parameters should be evaluated in routine orthopedics practice. Hence, we conducted this scoping review and meta-analysis to address these gaps in the existing literature.

## Review

Methods

This scoping review and meta-analysis was conducted following the Preferred Reporting Items for Systematic Reviews and Meta-Analysis (PRISMA) extension for Scoping Review guidelines (PRISMA-ScR) [4]. The review protocol was formulated following the PRISMA for systematic review protocols (PRISMA-P) guidelines [5] and published a-priori [6].

Study Objectives

The primary objective of this review was to identify the geometrical parameters that describe the three-dimensional anatomy of the talar neck, the measurement techniques, and precision. As a secondary objective, the review also aimed to identify the normative values and geographic differences of these parameters.

Eligibility Criteria

Original research studies (clinical or cadaveric, prospective or retrospective, comparative or noncomparative), evaluating the geometry or morphology of the human talar neck were included. There were no restrictions on the language of publication. Studies that did not describe the talar neck geometry, animal studies, review articles, conference abstracts, and case reports were excluded [6].

Information Sources

The primary search was conducted on the PubMed, Embase, and Scopus databases, using a pre-specified search strategy. The searches were filtered to include only human studies (Table 1). For the secondary search, a manual search of references from the full text of all included articles and relevant review articles was done. There were no restrictions on the language or date of publication [6].

**Table 1:** The detailed primary search methodology

Database	Search String	Results
PubMed (Searched on 18^th^ February 2024)	("talus"[MeSH Terms] OR "talus"[All Fields] OR "tali"[All Fields] OR ("talus"[MeSH Terms] OR "talus"[All Fields] OR "astragalus"[All Fields] AND ("geometries"[All Fields] OR "geometry"[All Fields] OR ("geometric"[All Fields] OR "geometrical"[All Fields] OR "geometrically"[All Fields] OR "geometrics"[All Fields]) OR ("geometric"[All Fields] OR "geometrical"[All Fields] OR "geometrically"[All Fields] OR "geometrics"[All Fields]) OR ("morphometric"[All Fields] OR "morphometrical"[All Fields] OR "morphometrically"[All Fields] OR "morphometrics"[All Fields]) OR ("morphometries"[All Fields] OR "morphometry"[All Fields]) OR ("measurability"[All Fields] OR "measurable"[All Fields] OR "measurably"[All Fields] OR "measure s"[All Fields] OR "measureable"[All Fields] OR "measured"[All Fields] OR "measurement"[All Fields] OR "measurement s"[All Fields] OR "measurements"[All Fields] OR "measurer"[All Fields] OR "measurers"[All Fields] OR "measuring"[All Fields] OR "measurings"[All Fields] OR "measurment"[All Fields] OR "measurments"[All Fields] OR "weights and measures"[MeSH Terms] OR ("weights"[All Fields] AND "measures"[All Fields]) OR "weights and measures"[All Fields] OR "measure"[All Fields] OR "measures"[All Fields]) OR ("biometries"[All Fields] OR "biometry"[MeSH Terms] OR "biometry"[All Fields]) OR (("three"[All Fields] OR "threes"[All Fields]) AND ("dimensional"[All Fields] OR "dimensionalities"[All Fields] OR "dimensionality"[All Fields] OR "dimensionalized"[All Fields] OR "dimensionally"[All Fields])) OR (("three"[All Fields] OR "threes"[All Fields]) AND ("dimensional"[All Fields] OR "dimensionalities"[All Fields] OR "dimensionality"[All Fields] OR "dimensionalized"[All Fields] OR "dimensionally"[All Fields]) AND ("model"[All Fields] OR "models"[All Fields] OR "modeled"[All Fields] OR "modeler"[All Fields] OR "modeler s"[All Fields] OR "modelers"[All Fields] OR "modeling"[All Fields] OR "modelings"[All Fields] OR "modelization"[All Fields] OR "modelizations"[All Fields] OR "modelize"[All Fields] OR "modelized"[All Fields] OR "modelled"[All Fields] OR "modeller"[All Fields] OR "modellers"[All Fields] OR "modelling"[All Fields] OR "modellings"[All Fields] OR "models"[All Fields])))	2727
EMBASE (Searched on 18^th^ February 2024)	(geometry OR morphometry OR measurement OR biometry OR 'three-dimensional imaging') AND (talus OR astragalus OR tali)	1090
SCOPUS (Searched on 18^th^ February 2024)	( ( TITLE-ABS-KEY ( *talus* ) OR TITLE-ABS KEY ( *tali* ) OR TITLE-ABS KEY ( *astragalus* ) ) ) AND ( ( TITLE-ABS-KEY ( *geometry* ) OR TITLE-ABS-KEY ( *morphometry* ) OR TITLE-ABS-KEY ( *measurement* ) OR TITLE-ABS-KEY ( *morphology* ) OR TITLE-ABS-KEY ( *biometry* ) OR TITLE-ABS-KEY ( *three* AND *dimensional* AND *modeling* ) ) )	2523

Study Selection of Sources of Evidence

Two authors (SS and KJ) independently screened the titles and abstracts of all articles identified in the initial search, following which full texts of the shortlisted articles were obtained. Two authors (SS and SP) independently assessed the shortlisted full-text articles for inclusion into the review, according to the pre-specified inclusion/exclusion criteria. Discrepancies were resolved by consensus. Reasons for the exclusion of those studies for which full-text was obtained were documented. A reference management software (Zotero version 5.0.93; Corporation for Digital Scholarship, Vienna, Virginia, United States) was used to manage references.

Data Charting Process and Data Items

Two authors (SS and KJ) extracted the data from included studies on pre-piloted data collection forms; a third author (SP) verified the data for accuracy.

Outcome Measures

The outcome measures included talar neck length (NL), neck width (NW), neck height (NH), cross-sectional area (CSA) of the talar neck, declination angle (DA), inclination angle (IA), and torsion angle (TA). Methods of measurement were ascertained. Intra-class correlation coefficients (ICC) for each parameter were recorded, if these were reported by the study authors. To determine the variability in the measurement of the geometrical parameters by different measurement techniques, the coefficient of variance was determined. The coefficient of variance is a ratio of the standard deviation to the mean; it can also be expressed as a percentage. Lower values indicate better precision in measurement, and vice-versa. For this study, the %age CV was arbitrarily graded as ‘low’ if it was 30% and ‘high’ if it was >30%.

Critical Appraisal of Individual Sources of Evidence

The methodological quality of the studies included in the review was assessed by the National Institutes of Health (NIH) Case Series tool. This is a nine-item checklist; each item of the checklist is graded as ‘Yes’, ‘No’, ‘Not Reported (NR)’, ‘Cannot Determine (CD)’ or ‘Not Applicable (NA)’. For this review, a study was graded as ‘Good’ if the answer to all items in the checklist (excluding the not applicable items) was ‘Yes’. A ‘Fair’ grading was given if the answer to one or two items in the checklist was ‘No’ or ‘Not Reported’. If the answer to three or more items in the checklist was ‘No’ or ‘Not Reported’, the study was graded as ‘Poor’.

Synthesis of Results

For qualitative analysis, appropriate tables and data visualization diagrams were constructed. Quantitative analysis was done for the following parameters: (a) NL, (b) NW, (c) NH, (d) IA, (e) DA, and (f) TA. Pooled values of each of these parameters were estimated by the random-effects meta-analysis model [7]; values were reported as means with 95% confidence intervals. Forest plots were constructed to visualize the results. Statistical heterogeneity was evaluated by the I^2^ test. Leave-one-out sensitivity analysis was performed if high statistical heterogeneity was identified (I^2^ > 75%). No subgroup analysis or meta-regression was planned a priori, as we were uncertain about the nature of the data that would be extracted. However, after data extraction, subgroup analysis using a random-effects model was performed based on ethnicity; studies were classified as either ‘Asian’ or ‘Non-Asian’ for this purpose. Analysis of 95% confidence intervals (CI) was done to determine statistically significant differences in the geometrical parameters between the two subgroups. A parameter was deemed to be significantly different if the 95% CIs of the pooled estimate of the two groups did not overlap, and vice versa. Analyses were performed by the OpenMeta[Analyst] (Brown University, Rhode Island, United States) and Stata Statistical Software MP version 14.0 (StataCorp LLC, College Station, Texas, United States) [6].

Results

Literature Search

**Figure 1:**
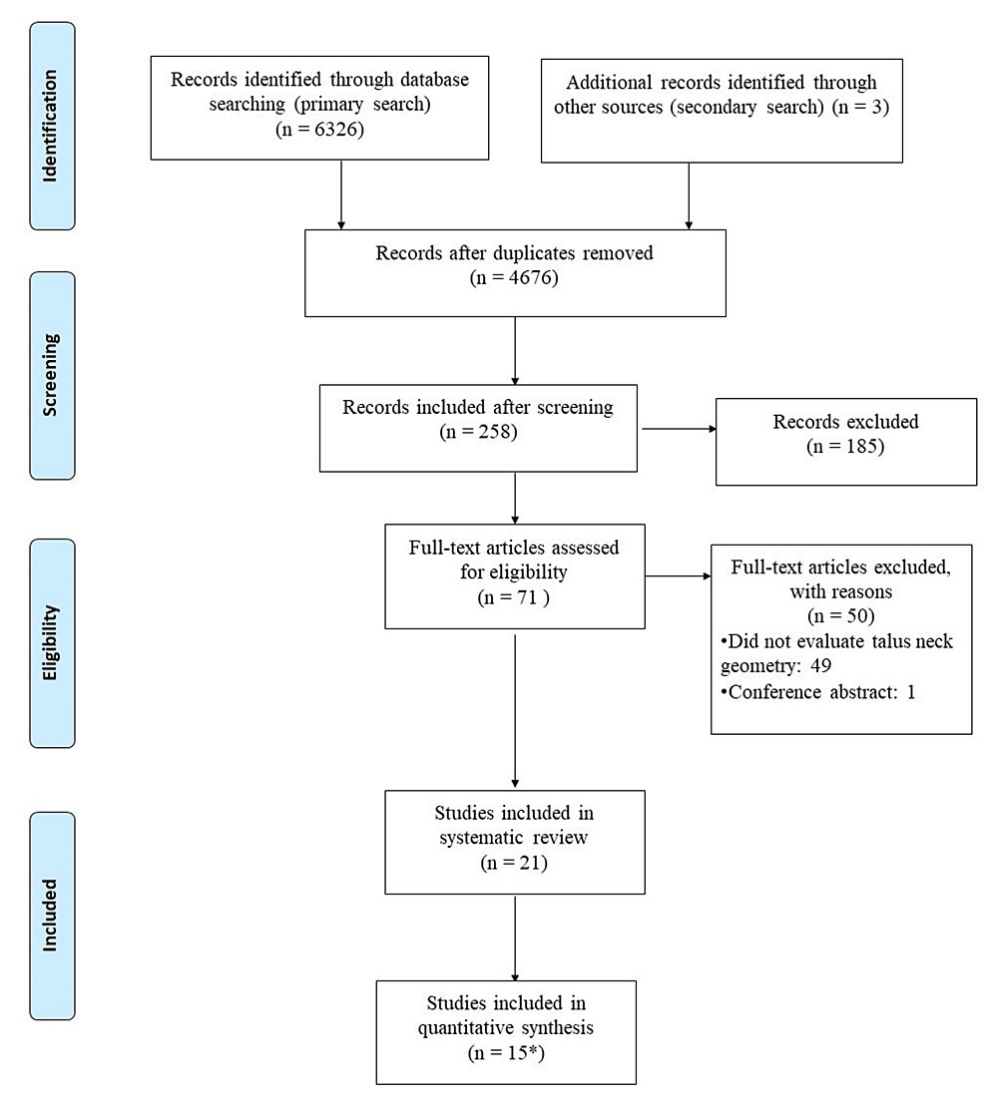
PRISMA Flow Diagram for the study PRISMA: Preferred Reporting Items for Systematic Reviews and Meta-Analysis

The combined search revealed 6326 results. After screening the titles and abstracts, 71 full-text articles were obtained. Of these, 21 were included in the scoping review [8-28]. Fifteen articles were included in six different sets of pooled meta-analyses to calculate the normative values of talar NL, NW, NH, DA, IA, and TA (Figure 1).

Of the 50 full-text articles excluded, the majority (n=49, 98%) did not report talar neck geometry as an outcome measure, and one was a conference abstract (Figure 1).

Characteristics of Studies Included

Of the 21 studies included in the review, seven (33.3%) included data from human subjects, whereas the other 14 (67.3%) were cadaveric. The majority of the studies (n=19, 90.5%) evaluated adult tali, and only two (9.5%) evaluated pediatric tali. In most of the studies (n=13, 61.9%), talus neck geometry was evaluated directly on dry bones or anatomical specimens; evaluation by imaging techniques (radiographs, CT, MRI, and radio-stereometric analysis) was used in other studies (Table 2).

**Table 2:** Baseline characteristics of studies included in the review ^*^tali were obtained from patients undergoing lower limb amputation A: adult subjects; P: pediatric subjects; C: cadaveric study; H: study conducted on alive human subjects/patients; IA: inclination angle, DA: declination angle; TA: torsion angle; NL: neck length; NW: neck width; NH: neck height; CSA: cross sectional area; RSA: radiostereometric analysis; MIND: minimum intra-articular distance of the neck

SNo	Author	Journal	Year	Type	Age	Country	Total Tali	Males	Females	Mean Age	Measurements made on	Talar Neck Parameter(s) evaluated
1	Abd-Elaleem et al. [10]	J Leg Med	2012	C	A	Egypt	110	67	43	Not recorded	Bones	NL, NW, NH
2	Agoada [8]	Anat Rec	2018	H*	A	United States	54	32	19	Not mentioned	Bones & radiographs	NL, NW
3	Agoada and Kramer [16]	JAPMA	2019	H*	A	United States	54	32	22	Not mentioned	Bones & radiographs	DA, IA
4	Bonnel et al. [17]	Orthop Traumat Surg Res	2011	C	A	France	40	Not mentioned	Not mentioned	Not mentioned	Bones	DA, IA, TA
5	Chan et al. [26]	J Orthop Trauma	2008	C	A	Canada	8	8	0	71 years	Radiographs, CT & RSA	Fracture displacement
6	Ebraheim et al. [14]	Foot Ankle Int	1996	C	A	United States	50	32	17	Not mentioned	Bones	NW, NH (lateral talar neck)
7	Farsetti et al. [18]	CORR	2009	H	A	Italy	72 patients	52	20	Not mentioned	CT	DA
8	Harnroongrooj et al. [9]	Thai J Surg	2003	C	A	Thailand	110	22	35	Not mentioned	Bones	NL, NW, NH
9	He et al. [15]	Orthop Surg	2016	C	A	China	130	33	32	43.9 years	CT	NH, CSA, DA, IA, TA
10	Ippolito et al. [19]	AJR	2004	H	A	Italy	48 clubfeet and 28 normal feet	28	10	25 years	CT	DA
11	Itohara et al. [20]	Eur J Rad	2005	H	P	Japan	10	2	3	5 months	MRI	DA, IA
12	Lee et al. [13]	J For Sci	2011	C	A	Korea	140	70	70	Not mentioned	Bones	NL
13	Mahakkanukrauh et al. [28]	Foren Sci	2014	C	A	Thailand	252	156	156	66 years	Bones	MIND
14	Mahato and Murthy [24]	Foot	2012	C	A	India	52	Not mentioned	Not mentioned	Not mentioned	Bones	DA, TA
15	Mahato [21]	Foot	2011	C	A	India	52	Not mentioned	Not mentioned	Not mentioned	Bones	DA, IA, TA
16	Motagi [23]	Int J Med Sci Pub Health	2014	C	A	India	52	Not mentioned	Not mentioned	Not mentioned	Bones	DA, IA
17	Nozaki et al. [22]	Surg Rad Anat	2017	C	A	Japan	50	50	0	36 years	Bones	DA, IA, TA
18	Nozaki et al. [27]	Clin Anat	2020	H	A	Japan	56	31	25	49.2 years	CT	Statistical Shape Modelling
20	Peckman et al. [11]	J Leg Med	2015	C	A	Greece	182	96	86	Not mentioned	Bones	NL
21	Ramalho et al. [25]	Rev Bras Ortop	1996	H	P	Spain	94	Not mentioned	Not mentioned	Not mentioned	Bones	DA
22	Bidmos and Dayal [12]	Foren Sci Int	2018	C	A	South Africa	220	110	110	Not mentioned	Bones	NL

**Figure 2:**
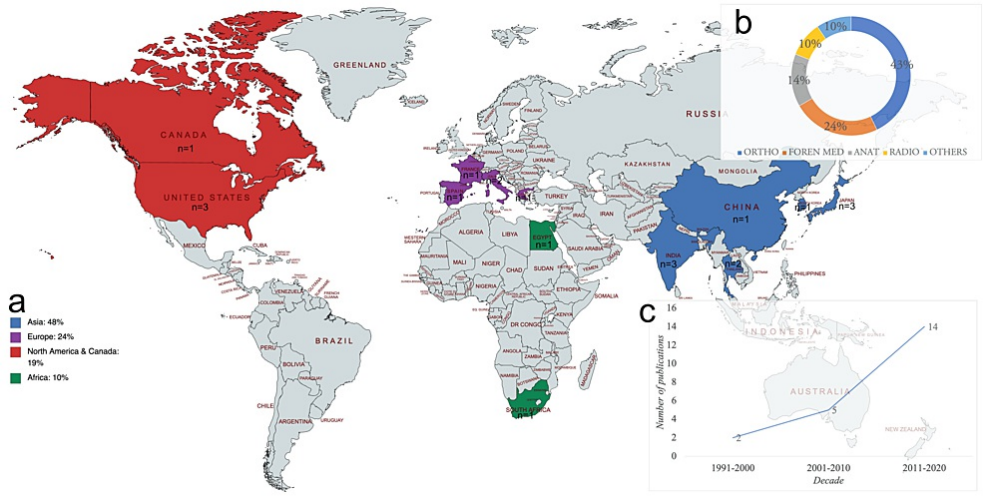
Baseline characteristics of studies included in the review (a) Geographical distribution, (b) Journal of publication, (c) Time trends. Ortho: orthopedics; Foren Med: Forensic Medicine; Anat: Anatomy; Radio: radiology Image Credit: Authors

Of the studies, 48% (n=10) were from Asia, 24% (n=5) from Europe, 19% (n=4) from North America and Canada, and 10% (n=2) from Africa. The majority (66%, n=14) of the studies were published in the recent decade (2011-2020) (Figure 2).

Talar NL

**Figure 3:**
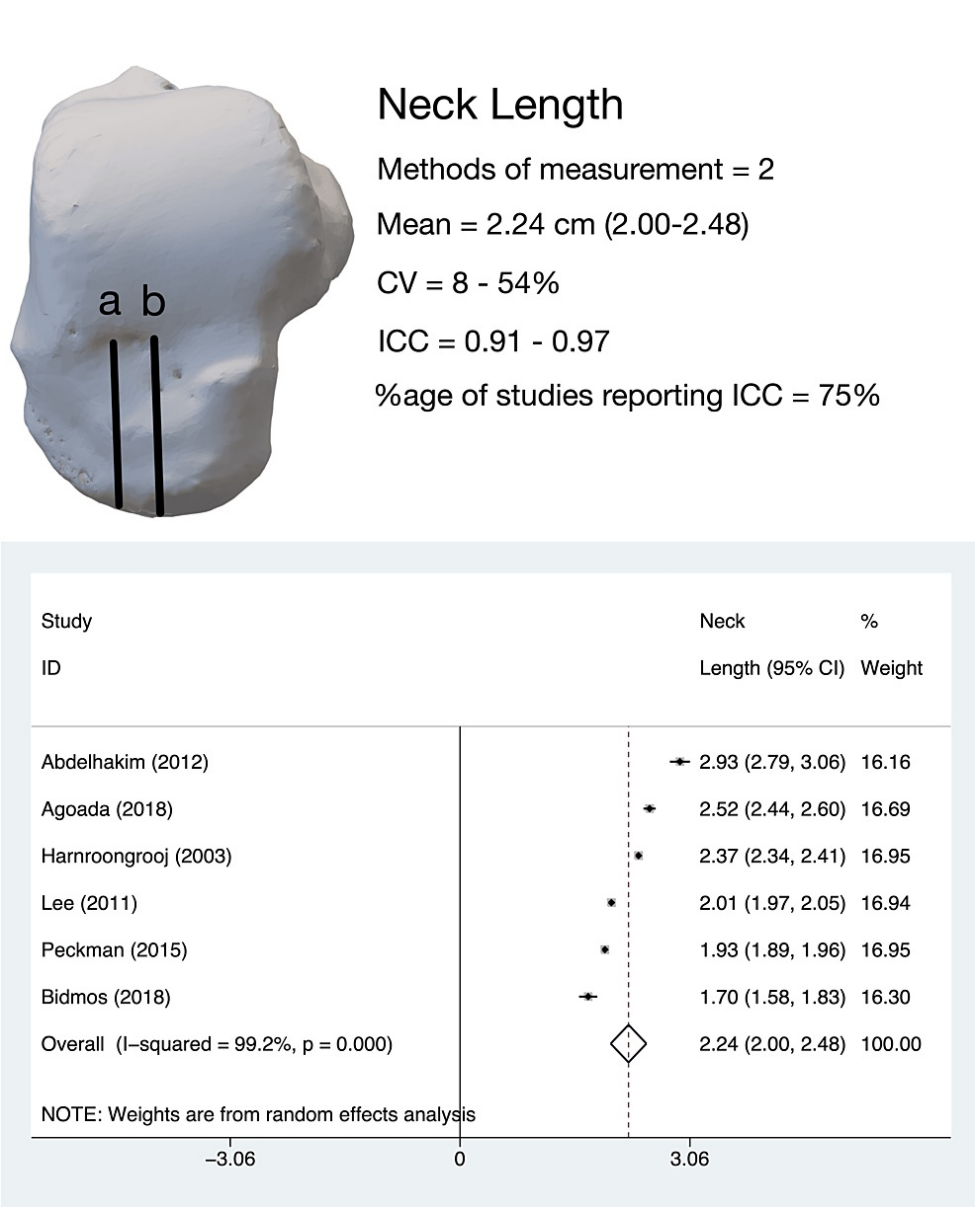
Talar neck length: methods of measurement, variability in measurement techniques and pooled meta-analysis estimates. Talar neck length measurement methods: (a) the method described by Bidmos and Dayal [12], Lee et al. [13], Peckmann et al. [11], Agoada et al. [8], Harnroongroj et al. [9]; (b) the method described by Abd-Elaleem et al. [10] CV: coefficient of variation; ICC: intra-class correlation coefficient

The anteroposterior dimension of the dorsal aspect of the talar neck in the horizontal plane, termed variably as ‘neck length’, ‘head-neck length’, or ‘maximum length of head-neck of talus’, was evaluated by six studies [8-13]. The pooled estimate of talar NL (six studies, 816 tali) was 2.24 cm (95%CI, 2.00-2.48); the heterogeneity for this estimate was high (I^2^ = 99.2%) (Figure 3).

Leave-one-out sensitivity analysis revealed that the heterogeneity was not altered by the omission of any study. Subgroup analysis revealed that the non-Asian population had a marginally (insignificantly) higher NL (2.269 cm, 95%CI, 1.813-2.724) than the Asian population (2.194 cm, 95%CI, 1.841-2.547). Except for two studies (Abd-elhakim et al. [10] 24%, Bidmos et al. [12] 54%), the coefficient of variance for this parameter was determined to be low.

Talar NW

**Figure 4:**
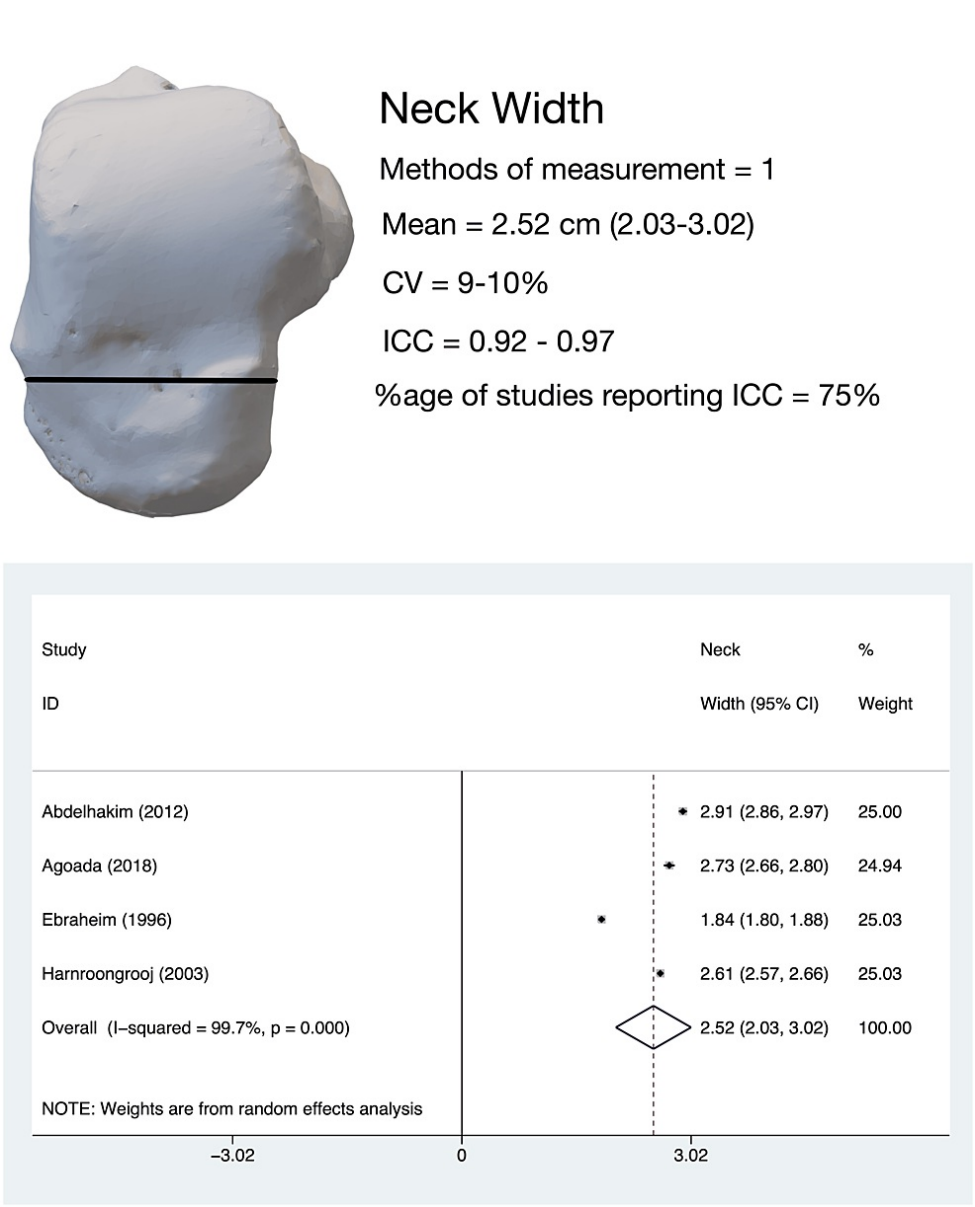
Talar neck width: methods of measurement, variability in measurement techniques and pooled meta-analysis estimates. All four studies [8-10,13,14] evaluating talar width used the same measurement technique. All meta-analysis values are in centimeters. CV: coefficient of variation; ICC: intra-class correlation coefficient

The mediolateral dimension of the dorsal aspect of the talar neck in the horizontal plane, termed ‘neck width’, or ‘minimum width of the neck of talus’, was evaluated by four studies [8-10,13,14]. The pooled estimate of talar width (four studies, 324 tali) was 2.52 cm (95%CI, 2.03-3.02); the heterogeneity for this estimate was high (I^2^ = 99.7%) (Figure 4).

Leave-one-out sensitivity analysis revealed that the heterogeneity was not altered by the omission of any study. Subgroup analysis revealed that the Asian population had a marginally (insignificantly) higher NW (2.614 cm, 95%CI 2.571-2.656) than the non-Asian population (2.494 cm, 95%CI, 1.765-3.224). The coefficient of variance for this parameter was determined to be low for all studies.

Talar NH

**Figure 5:**
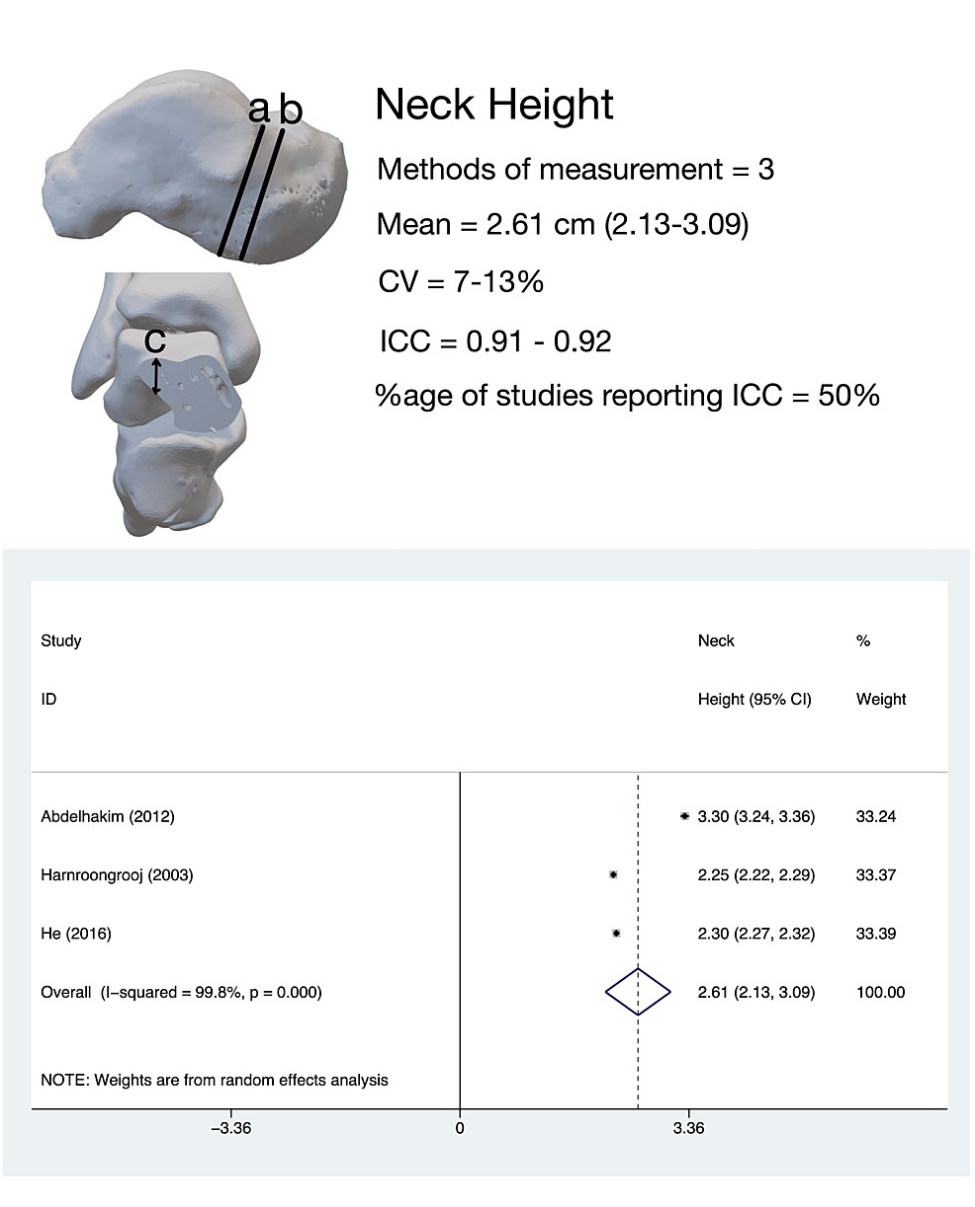
Talar neck height: methods of measurement, variability in measurement techniques, and pooled meta-analysis estimates. Talar neck height measurement methods: (a) the measurement method for medial talar neck height as described by He et al. [15] and Harnroongroj et al. [9]; (b) the measurement method for medial talar neck height as described by Abd-Elaleem et al. [10]; (c) the measurement method for lateral talar neck height described by Ebraheim et al. [14]. All meta-analysis values are in centimeters. CV: coefficient of variation; ICC: intra-class correlation coefficient

The vertical dimension of the talar neck in the sagittal plane, termed ‘neck height’, or ‘neck thickness’, was evaluated by four studies [9,10,14,15]. The pooled estimate of medial talar NH (three studies, 350 tali) was 2.62 cm (95%CI, 2.14-3.09); the heterogeneity for this estimate was high (I^2^ = 99.7%) (Figure 5).

Leave-one-out sensitivity analysis revealed that the heterogeneity was not altered by the omission of any study. Subgroup analysis revealed that the Non-Asian population had a significantly higher medial neck height (3.301 cm, 95%CI, 3.239-3.363) than the Asian population (2.274 cm, 95%CI, 2.231-2.317). The coefficient of variance for this parameter was determined to be low for all studies. Ebraheim et al. [14] determined the thickness (height) of various parts of the lateral talar neck (Figure 5). It was found to increase progressively from lateral to medial and was noted to have a maximum value of 8 mm medial to the lateral border.

DA

**Figure 6:**
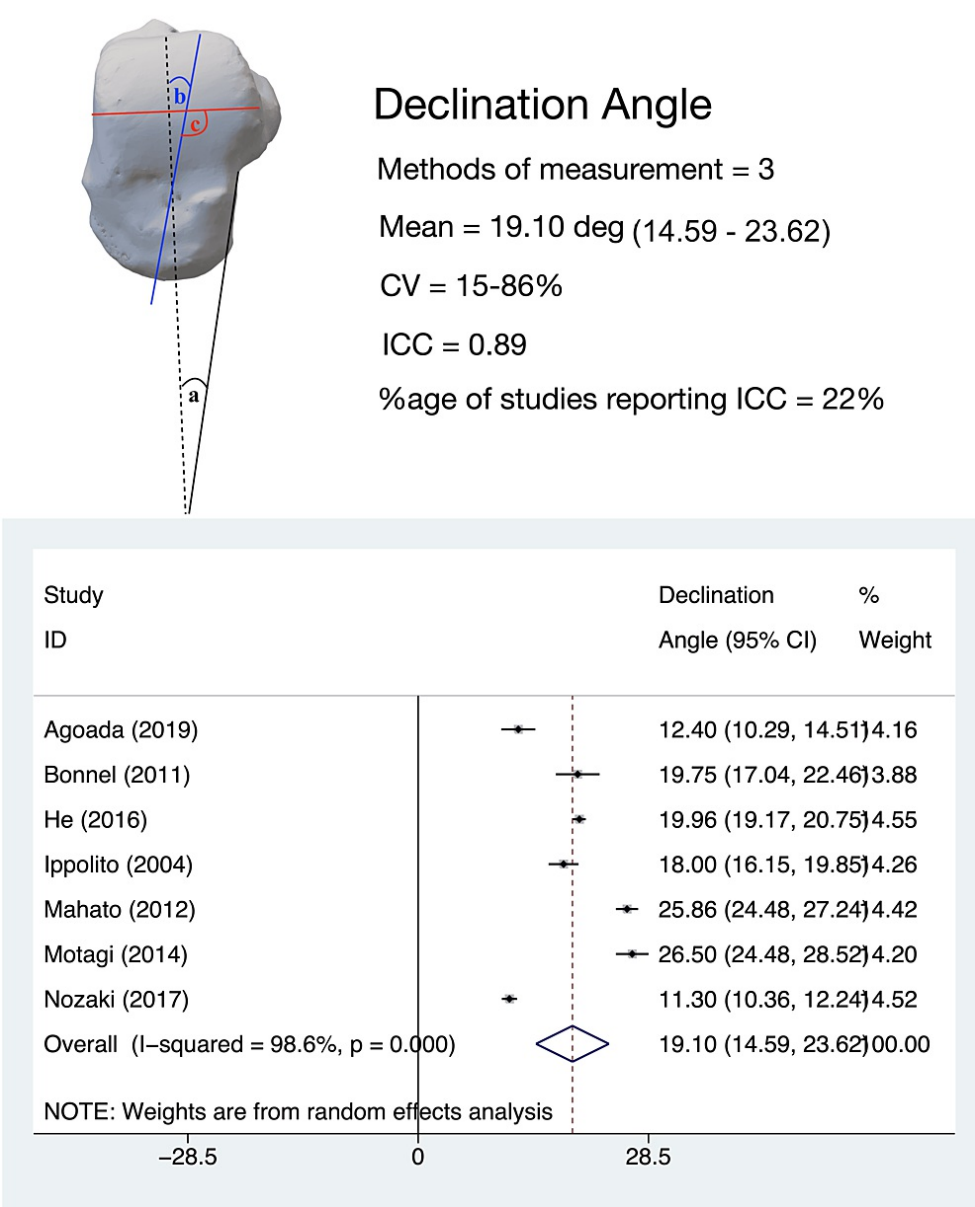
Talar neck declination angle: methods of measurement, variability in measurement techniques and pooled meta-analysis estimates. Declination angle measurement methods:  (a) the measurement method described by Agoada et al. [8]; (b) the measurement method described by Bonnel et al. [17], He et al. [15], Ippolito et al. [19], and Nozaki et al. [22]; (c) the measurement method described by Mahato et al. [21] and Mahato et al. [24]. All meta-analysis values are in degrees. CV: coefficient of variation; ICC: intra-class correlation coefficient

The angle between the body and neck of the talus in the horizontal plane, termed variably as ‘declination angle’, ‘medial neck angle’, ‘horizontal angle of talar neck’, ‘transverse angle of talar head’, ‘talar body-neck-head angle’ or ‘varus angle of the neck’, was evaluated by nine studies [15-23]. While the majority of these studies measured this angle between the long axis of the talar body and the long axis of the talar neck, Mahato et al. [21,24] measured this between the transverse axis of the talar body and the long axis of the talar neck. The pooled estimate of DA in adults (seven studies, 406 tali) was 19.10° (95%CI, 14.59-23.62); the heterogeneity for this estimate was high (I^2^ = 98.6%) (Figure 6).

Leave-one-out sensitivity analysis revealed that the heterogeneity was not altered by the omission of any study. Subgroup analysis revealed that the Asian population had a higher DA (20.873°, 95%CI, 14.216-27.530) than the Non-Asian population (16.672°, 95%CI, 12.414-20.931); however, this difference was not statistically significant. The coefficient of variance for this parameter was determined to be high for three studies [16,17,20], and low for others. Two studies evaluated the DA in the pediatric population [20,25]. Ramalho et al. showed that the DA progressively decreases with the growth of the foot [25]. In an MRI-based study on infants with unilateral clubfoot, Itohara et al. showed that tali in clubfeet had a significantly higher DA, lower total volume, and lower volume of the ossific nucleus as compared to tali in the contralateral, normal foot [20].

IA

**Figure 7:**
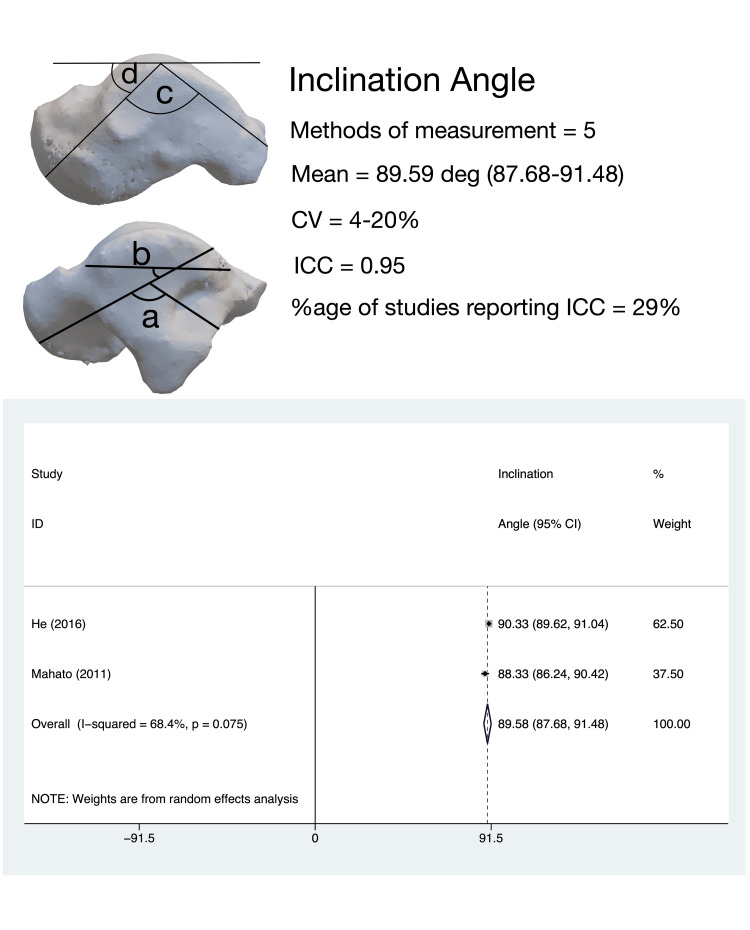
Talar neck inclination angle: methods of measurement, variability in measurement techniques and pooled meta-analysis estimates. Inclination angle measurement methods: (a) the method described by Bonnel et al. [17]; (b) the measurement method described by Agoada et al. [16]; (c) the method described by He et al. [15] and Mahato et al. [21]; (d) the method described by Motagi et al. [23]. All meta-analysis values are in degrees. CV: coefficient of variation; ICC: intra-class correlation coefficient

The angle between the body and neck of the talus in the sagittal plane, termed variably as ‘inclination angle’, ‘vertical neck angle’, ‘plantar deviation of talar head’, and ‘sagittal angle of talar head’ was evaluated by 10 studies [15-24]. Six different measurement techniques were used for measuring this angle; only two studies [15,21] used similar measurement techniques. The pooled estimate of IA in adults (two studies, 182 tali) was 89.58° (95%CI, 87.68-91.48); heterogeneity for this estimate was moderate (I^2^ = 68.4%) (Figure 7).

The coefficient of variance for this parameter was determined to be low for all studies.

TA

**Figure 8:**
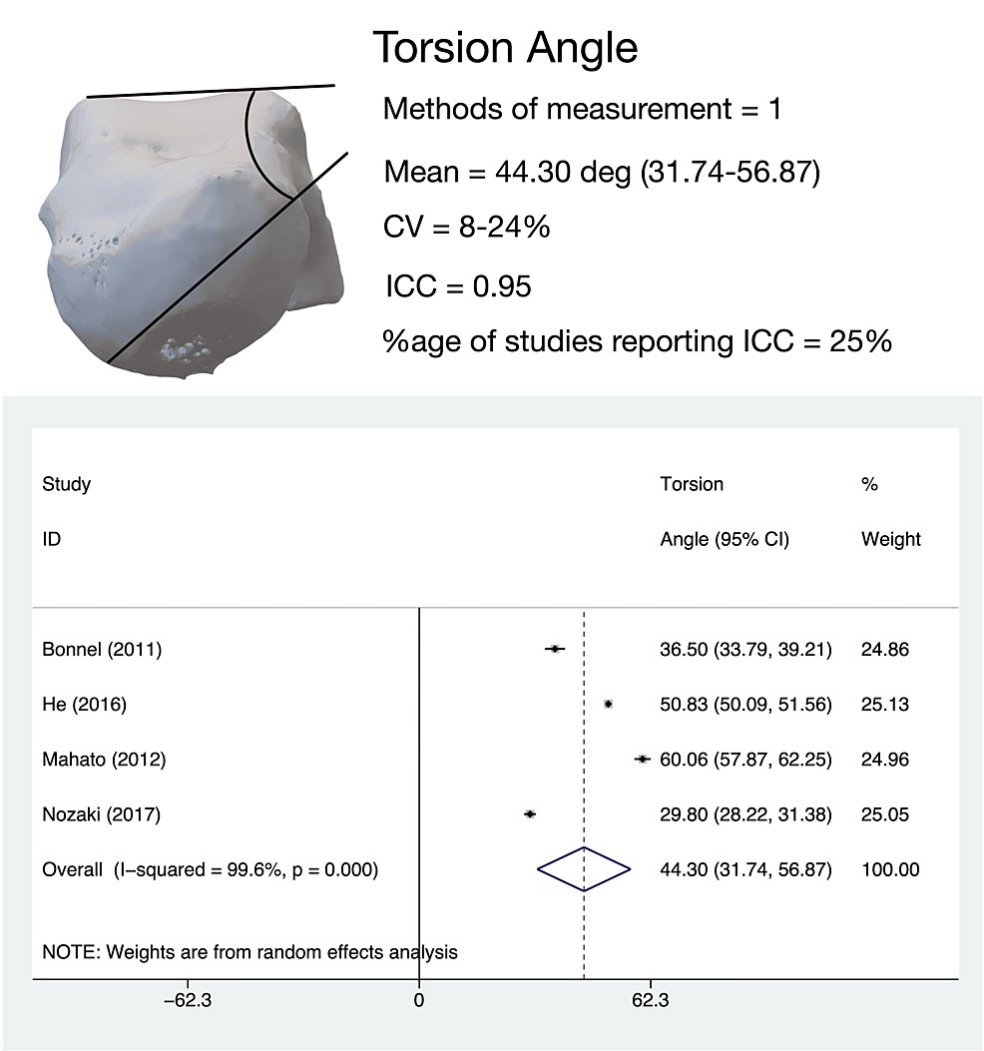
Talar neck torsion angle: methods of measurement, variability in measurement techniques and pooled meta-analysis estimates. All four studies [15,17,22,24] evaluating talar torsion used the same measurement technique. All meta-analysis values are in degrees. CV: coefficient of variation; ICC: intra-class correlation coefficient

The angle between the head and body of the talus in the coronal plane, termed variably as ‘rotation angle’, ‘torsion angle’, or ‘coronal plane angle’ was evaluated by four studies [15,17,22,24]. All four studies employed similar measurement techniques for this parameter. The pooled estimate of TA angle in adults (four studies, 272 tali) was 44.3° (95%CI, 31.74-56.87); heterogeneity for this estimate was high (I^2^ = 99.6%) (Figure 8). Leave-one-out sensitivity analysis revealed that the heterogeneity was not altered by the omission of any study.

Subgroup analysis revealed that the Asian population had a higher TA (46.884°, 95%CI 31.668-62.1) than the Non-Asian population (36.5°, 95%CI 33.788-39.212); however, this difference was not statistically significant. The coefficient of variance for this parameter was determined to be low for all studies.

Other Parameters

He et al. determined the cross-sectional area and circumference of the talar neck [15]. The mean area was 14.80 ± 2.02 cm^2^, whereas the circumference was 8.92 ± 0.6 cm. In a cadaveric study, Chan et al. compared conventional radiographs, CT scans, and radio stereometric assay (RSA) to malreduction after fixation of talar neck fractures [26]. While all techniques underestimated malreduction, CT was the most accurate of all three techniques. Nozaki et al. performed a three-dimensional geometric morphometric analysis to determine differences in talar geometry in men and women [27]. Women were noted to have a longer talar neck and a higher TA, as compared to men.

Critical Appraisal within Sources of Evidence

Only three of the 21 (14.3%) studies included in the review were deemed to be of ‘Good’ methodological quality as per the NIH Case Series tool. Eleven (52.4%) studies were graded as ‘Fair’ whereas the remaining seven (33.3%) were graded as ‘Poor’ (See Appendices). Reasons for poor methodology included lack of comparability of the study cohort (higher percentage of males as compared to females), use of measurement methods prone to high inter and intra-observer variability, as well as instrument error (goniometer and vernier calipers), and ill-defined statistical methods.

Discussion

Talar neck fractures are often associated with comminution on the medial side, which results in shortening of the medial talar neck, and consequently, a varus deformity [29-35]. These fractures necessitate anatomical reduction, as even small amounts of varus malalignment can impact the subtalar joint and midfoot biomechanics. Sangeorzan et al. have shown that a 2 mm displacement of the talar neck can significantly affect the contact characteristics of the subtalar joint [2]. Furthermore, Daniels et al. have demonstrated that a varus malalignment of the talar neck can result in varus and internal rotation deformity of the hindfoot, adduction deformity of the forefoot, and limitation of eversion [3]. However, assessment of varus after fixation of talar neck fractures remains challenging, and requires bilateral visualization of the talar neck margins [32,33]. In 1978, Canale and Kelly described the ‘Canale view’ to image the talar neck in the horizontal plane, and to detect varus malalignment [34]. This view allows for visualization of the medial comminution of the talar neck and discontinuity of the lateral cortical margin indicative of a varus malreduction. This view is performed with the ankle maximally plantarflexed, the foot pronated by 15°, and the X-ray beam angled 75° cephalad. Thomas and Boyce suggested that varus malreduction may be appreciated better by using multiple Canale views, with the pronation varying from 15° to 25° [35]. Given the fact that the surgeon must aim to achieve < 2mm of displacement of the talar neck after reduction, quantification of the reduction assumes paramount importance. As Bonnel et al. have shown the normal alignment of the talar neck in the horizontal plane can be varus, neutral, or even valgus, it is important to know and quantify the normal anatomy before the surgeon can plan operative fixation or correction of malunion for talar neck fractures [17]. The DA can be used for this purpose, with the normal, contralateral side serving as the target.

Correction of a talar neck malunion necessitates a talar neck osteotomy and restoration of the medial neck length employing an interposition graft. The few published studies on talar neck malunion [36-40], while reporting the ‘varus deformity’, did not quantify its three-dimensional anatomy nor report the correction that was achieved post-operatively. We believe that the DA and TA are useful parameters to quantify the degree of varus and internal rotation of the neck in a talar malunion respectively. Measurement of these parameters on the normal, contralateral limb can also provide the surgeon with an estimate of the degree of correction that is needed.

The orthopedic literature also reports dorsiflexion malunion after talar neck fractures [34,40]. In these cases, the distal fragment displaces or angulates in a dorsal direction. The net effect is decreased ankle dorsiflexion owing to impingement of the dorsally displaced talar head. Whereas dorsal displacement is easy to assess on lateral ankle radiographs, dorsal tilt can be hard to judge intra-operatively. Hence, we believe that assessment of the IA, and comparison with the uninjured contralateral side can serve as a reliable guide to sagittal plane reduction of the talar neck. A summary of these parameters has been provided in Table 3.

**Table 3:** Pooled values and clinical significance of different parameters evaluated in the review.

SNo.	Characteristic	Synonyms	Pooled Value (95% CI)	Significance
1	Neck Length	Head-Neck Length	2.24 cm (2-2.48 cm)	Differential shortening of the medial neck length, as compared to the lateral neck length results in varus. Equal loss of medial and lateral neck lengths results in foreshortening of talus
2	Neck Width	-	2.52 cm (2.03-3.02 cm)	Unclear
3	Neck Height	Neck Thickness	2.61 cm (2.13-30.9 cm)	Lateral height is important for posterior to anterior screws
4	Frontal Plane Angle	Declination Angle/Varus Angle of Neck	19.1 degrees (14.59-23.62 degrees)	Increase in this angle is suggestive of varus malunion
5	Sagittal Plane Angle	Inclination Angle/Vertical Neck Angle	89.59 degrees (87.68-91.48 degrees)	Dorsiflexion malunion
6	Torsion Angle	Rotational Angle	44.3 degrees (31.74-56.87 degrees)	Talar malunions show alterations in the torsion angle, in addition to an increased frontal plane angle

When performing this review, we noted that most of the published literature on the three-dimensional geometry of the talar neck comes from anthropological studies. These studies report on a wide variety of parameters of the talar head, neck, body, and articular surfaces. The focus of these studies was to map anthropology and predict biomechanics, design implants for ankle arthroplasty or total talar replacement, or, in forensic medicine, to predict gender. We did not find any study evaluating the restoration of talar neck geometry during or after operative fixation of talar neck fractures. In contrast to other foot and ankle fractures, the orthopedic literature does not report on talar neck geometry quantitatively in talar neck fractures or malunions. We hope that this review provides the impetus for future studies that aim to quantify the post-operative reduction in talar neck fractures on radiographs and CT scans. Further research should focus on the measurement of these parameters on conventional, as well as volume-rendered CT and MRI sections.

This review has several strengths such as strict adherence to the PRISMA-SCR guidelines, a well-defined search strategy covering multiple databases, sensitivity as well as subgroup analyses, and determination of the risk of bias. To the best of our knowledge, this is the first review to summarize the geometrical parameters to assess the talar neck. The pooled estimates of each geometrical parameter, estimates of the ICC, as well as the coefficient of variation of each of these parameters, are provided.

We acknowledge the inherent limitations of our review, which is based on a heterogeneous database. This is reflected in the high statistical heterogeneity of our pooled estimates. This may be attributed to geographical and ethnic differences, and also to the fact that different authors have used different imaging modalities and measurement techniques. Furthermore, many studies included in the review evaluated tali obtained from cadavers and amputation specimens, which could potentially have been afflicted by the disease. Hence, these results should be interpreted with caution. These limitations notwithstanding, the precision in our pooled estimates is reflected by the narrow CIs.

## Conclusions

Although several parameters have been described to assess the talar neck geometry in three dimensions, their use to assess talus neck reduction in the orthopedic literature is sparse. Three of these parameters, the DA, IA, and TA can be used to quantify the frontal, sagittal, and coronal plane reduction of the talar neck. However, there is a need to standardize these measurements on conventional CT/MRI sections for use in routine orthopedic practice. Furthermore, our subgroup analysis has also revealed that the Asian Population has a significantly higher NH than the non-Asian population. These estimates can help orthopedic surgeons in determining the normal range of values of these parameters and the variations that might be expected.

## Appendices

**Table 4:** Risk of bias assessment using the NIH case series tool ^1^ higher percentage of males; ^2^ measurements were made by vernier calipers or goniometer NIH: National Institutes of Health; Y: yes; N: no; N/A: not applicable; NR: not reported by the study authors; CD: Could not be determined from the study data

S. No.	Author	Was the study question or objective clearly stated?	Was the study population clearly and fully described, including a case definition?	Were the cases consecutive?	Were the subjects comparable?	Was the intervention (method of measurement) clearly described?	Were the outcome measures clearly defined, valid, reliable and implemented consistently across all study participants?	Was the length of follow-up adequate?	Were the statistical methods well described?	Were the results well described?	Overall Grading
	Abdelhakim et al., 2012 [10]	Y	Y	N/A	N^1^	Y	N^2^	N/A	Y	Y	Fair
	Agoada 2018, [8]	Y	Y	NR	N^1^	Y	Y	N/A	Y	Y	Fair
	Agoada and Kramer, 2019 [16]	Y	Y	NR	N^1^	Y	Y	N/A	Y	Y	Fair
	Bidmos and Dayal, 2018 [12]	Y	Y	N/A	Y	Y	N^2^	N/A	Y	Y	Fair
	Bonnel et al., 2011 [17]	Y	Y	N/A	NR	Y	Y	N/A	N	Y	Fair
	Chan et al., 2008 [26]	Y	Y	N/A	Y	Y	Y	N/A	Y	Y	Good
	Ebraheim et al., 1996 [14]	Y	Y	N/A	N^1^	Y	N^2^	N/A	N	Y	Poor
	Farsetti et al., 2009 [18]	Y	Y	CD	N^1^	Y	Y	N/A	Y	Y	Fair
	Harnroongrooj et al., 2019	Y	Y	N/A	N^1^	Y	N^2^	N/A	N	Y	Poor
	He et al., 2016 [15]	Y	Y	N/A	Y	Y	Y	N/A	Y	Y	Good
	Ippolito et al., 2004 [19]	Y	Y	Y	N^1^	Y	Y	N/A	N	Y	Fair
	Itohara et al., 2005 [20]	Y	Y	NR	Y	Y	Y	N/A	Y	Y	Fair
	Lee et al., 2011 [13]	Y	Y	N/A	Y	Y	N^2^	N/A	N	Y	Fair
	Mahakkanukrauh et al., 2014 [28]	Y	Y	N/A	Y	Y	N^2^	N/A	Y	Y	Fair
	Mahato, 2011 [21]	Y	Y	N/A	NR	Y	N^2^	N/A	N	Y	Poor
	Mahato and Murthy, 2012 [24]	Y	Y	N/A	NR	Y	N^2^	N/A	N	Y	Poor
	Motagi, 2014 [23]	Y	Y	N/A	NR	Y	N^2^	N/A	N	Y	Poor
	Nozaki et al., 2017 [22]	Y	Y	N/A	Y	Y	Y	N/A	Y	Y	Good
	Nozaki et al., 2020 [27]	Y	Y	NR	N^1^	Y	Y	N/A	Y	Y	Fair
	Peckman et al., 2015 [11]	Y	Y	N/A	N	Y	N^2^	N/A	N	Y	Poor
	Ramalho et al., 1996 [25]	Y	Y	NR	NR	Y	Y	N/A	N	Y	Poor

## References

[1] Buza JA 3rd, Leucht P (2018). Fractures of the talus: current concepts and new developments. Foot Ankle Surg.

[2] Sangeorzan BJ, Wagner UA, Harrington RM, Tencer AF (1992). Contact characteristics of the subtalar joint: the effect of talar neck misalignment. J Orthop Res.

[3] Daniels TR, Smith JW, Ross TI (1996). Varus malalignment of the talar neck. Its effect on the position of the foot and on subtalar motion. J Bone Joint Surg Am.

[4] Tricco AC, Lillie E, Zarin W (2018). PRISMA extension for scoping reviews (PRISMA-ScR): checklist and explanation. Ann Intern Med.

[5] Moher D, Shamseer L, Clarke M (2015). Preferred reporting items for systematic review and meta-analysis protocols (PRISMA-P) 2015 statement. Syst Rev.

[6] Sharma S, Jindal K, Patel S, Prabhakar S, Dhillon MS (2020). Parameters for assessment of talar neck geometry: a protocol for scoping literature review [PREPRINT]. medRxiv.

[7] DerSimonian R, Laird N (1986). Meta-analysis in clinical trials. Control Clin Trials.

[8] Agoada D (2018). The relationship between linear osteological and radiographic measurements of the human calcaneus and talus. Anat Rec (Hoboken).

[9] Harnroongroj T, Volpert LG, Ellis SJ, Sofka CM, Deland JT, Demetracopoulos CA (2019). Comparison of tibial and talar bone density in patients undergoing total ankle replacement vs non-ankle arthritis matched controls. Foot Ankle Int.

[10] Abd-elaleem SA, Abd-elhameed M, Ewis AA (2012). Talus measurements as a diagnostic tool for sexual dimorphism in Egyptian population. J Forensic Leg Med.

[11] Peckmann TR, Orr K, Meek S, Manolis SK (2015). Sex determination from the talus in a contemporary Greek population using discriminant function analysis. J Forensic Leg Med.

[12] Bidmos MA, Dayal MR (2003). Sex determination from the talus of South African Whites by discriminant function analysis. Am J Forensic Med Pathol.

[13] Lee UY, Han SH, Park DK, Kim YS, Kim DI, Chung IH, Chun MH (2012). Sex determination from the talus of Koreans by discriminant function analysis. J Forensic Sci.

[14] Ebraheim NA, Mekhail AO, Salpietro BJ, Mermer MJ, Jackson WT (1996). Talar neck fractures: anatomic considerations for posterior screw application. Foot Ankle Int.

[15] He JQ, Ma XL, Zhang X, Xin JY, Li N (2016). Three-dimensional computer-assisted modeling of talus morphology in chinese patients. Orthop Surg.

[16] Agoada D, Kramer PA (2019). The relationship between angular osteologic and radiographic measurements of the human talus and calcaneus. J Am Podiatr Med Assoc.

[17] Bonnel F, Teissier P, Maestro M, Ferré B, Toullec E (2011). Biometry of bone components in the talonavicular joint: a cadaver study. Orthop Traumatol Surg Res.

[18] Farsetti P, Dragoni M, Ippolito E (2012). Tibiofibular torsion in congenital clubfoot. J Pediatr Orthop B.

[19] Ippolito E, Fraracci L, Farsetti P, De Maio F (2004). Validity of the anteroposterior talocalcaneal angle to assess congenital clubfoot correction. AJR Am J Roentgenol.

[20] Itohara T, Sugamoto K, Shimizu N (2005). Assessment of the three-dimensional relationship of the ossific nuclei and cartilaginous anlagen in congenital clubfoot by 3-D MRI. J Orthop Res.

[21] Mahato NK (2011). Morphology of sustentaculum tali: biomechanical importance and correlation with angular dimensions of the talus. Foot (Edinb).

[22] Nozaki S, Watanabe K, Katayose M (2017). Three-dimensional morphometric analysis of the talus: implication for variations in kinematics of the subtalar joint. Surg Radiol Anat.

[23] Motagi M, Kottapurath S, Dharwadkar K (2015). Morphometric analyses of human dry tali of South Indian origin. Int J Med Sci Public Health.

[24] Mahato NK, Murthy SN (2012). Articular and angular dimensions of the talus: inter-relationship and biomechanical significance. Foot (Edinb).

[25] Ramalho Jr A, Kozonara ME, De Angelis MA, Jorge FF (1996). Anatomic study of the talus declination angle of normal children feet. Revista Brasileira de Ortopedia.

[26] Chan G, Sanders DW, Yuan X, Jenkinson RJ, Willits K (2008). Clinical accuracy of imaging techniques for talar neck malunion. J Orthop Trauma.

[27] Nozaki S, Watanabe K, Kamiya T, Katayose M, Ogihara N (2020). Three-dimensional morphological variations of the human calcaneus investigated using geometric morphometrics. Clin Anat.

[28] Mahakkanukrauh P, Praneatpolgrang S, Ruengdit S, Singsuwan P, Duangto P, Case DT (2014). Sex estimation from the talus in a Thai population. Forensic Sci Int.

[29] Rammelt S, Winkler J, Zwipp H (2013). Operative treatment of central talar fractures [Article in German]. Oper Orthop Traumatol.

[30] Whitaker C, Turvey B, Illical EM (2018). Current concepts in talar neck fracture management. Curr Rev Musculoskelet Med.

[31] Lee C, Brodke D, Perdue PW Jr, Patel T (2020). Talus fractures: evaluation and treatment. J Am Acad Orthop Surg.

[32] Rammelt S, Pitakveerakul A (2019). Hindfoot injuries: how to avoid posttraumatic varus deformity?. Foot Ankle Clin.

[33] Segura FP, Eslava S (2020). Talar neck fractures: single or double approach?. Foot Ankle Clin.

[34] Canale ST, Kelly FB (1978). Fractures of the neck of the talus. Long-term evaluation of seventy-one cases. J Bone Joint Surg Am.

[35] Thomas JL, Boyce BM (2012). Radiographic analysis of the Canale view for displaced talar neck fractures. J Foot Ankle Surg.

[36] Sakaki MH, Macedo RS, Godoy Dos Santos AL, Ortiz RT, Sposeto RB, Fernandes TD (2018). Talar body reconstruction for nonunions and malunions. Indian J Orthop.

[37] Suter T, Barg A, Knupp M, Henninger H, Hintermann B (2013). Surgical technique: talar neck osteotomy to lengthen the medial column after a malunited talar neck fracture. Clin Orthop Relat Res.

[38] Chen G, Hu M, Xu Y, Zhen YH, Hong Y, Xu XY (2017). Joint-preserving surgery for talar malunions or nonuions. Orthop Surg.

[39] Zwipp H, Gavlik M, Rammelt S (2014). Secondary anatomical reconstruction of malunited central talus fractures [Article in German]. Unfallchirurg.

[40] Rammelt S, Winkler J, Grass R, Zwipp H (2006). Reconstruction after talar fractures. Foot Ankle Clin.

